# An experimental manipulation of cognitive appraisals in parental burnout

**DOI:** 10.1038/s41598-023-38587-8

**Published:** 2023-07-18

**Authors:** Aline Woine, Dorota Szczygiel, Isabelle Roskam, Moïra Mikolajczak

**Affiliations:** 1 Department of Psychology, Psychological Sciences Research Institute, Université Catholique de Louvain (UCLouvain), Place Cardinal Mercier, 10, 1348 Louvain-la-Neuve, Belgium; 2 Department of Psychology, Faculty in Sopot, SWPS University, Sopot, Poland

**Keywords:** Psychology, Human behaviour

## Abstract

As it often applies to other mental conditions, one may posit that cognitive appraisals might be causal in the onset and maintenance of parental burnout. Recent studies have indeed highlighted that negative cognitive appraisals are positively associated with parental burnout. Howbeit, none of these studies being experimental in design, it has—thus far—been impossible to establish causality. To shed light on the question, the present study relied on an experimental design where the *perception* of three known antecedents of parental burnout was manipulated: co-parenting support, emotion regulation and child-rearing practices. 313 French- and English-speaking parents took part in the study which employed a 4 (Condition: control, perceived co-parenting support, perceived emotion regulation, perceived efficacy of child-rearing practices) × 2 (Time: pre- and post-manipulation) mixed-design, with Condition as the between-subject factor and Time as the within-subject factor. Results showed that the experimental manipulation was effective in the “co-parenting support” condition solely and this effective manipulation further yielded a significant effect on the decrease of parental burnout scores, hence suggesting a causative relation between cognitive appraisals and parental burnout. Our results highlight both the complexity of manipulating parents’ cognitive appraisals and the scope for relieving partnered parents from their parental burnout symptoms.

## Introduction

"There is nothing either good or bad but thinking makes it so", Hamlet, Act 2, Scene 2, 239-251. Not less than four centuries ago, when William Shakespeare wrote these famous lines, he serendipitously laid the foundation of a concept rooted in cognitive psychology theories since 1960’s: cognitive appraisal, that is, the subjective interpretation of an objective situation^1^. While the reader might consider the subtlety of English literature being rather far removed from the daily hassles of raising children, the following might help him/her reconsider. In a less literary but equally moving style, a twenty-first century anonymous exhausted father confides: “I tend to see the glass half empty when it comes to my children. My friends tell me that my children are no more difficult than anyone else's (...) My wife tells me that our children are doing well at school, that they are healthy and that they are just children. What I see is that they don’t seem to listen to me nor obey to me. It’s always a struggle. Every day the same struggle. I'm not doing well. I can't stand my children anymore. I simply can’t take it any more”.

Parental burnout is a syndrome characterized by a fourfold symptomatology arising from a prolonged overwhelming imbalance between parental resources (e.g., supportive co-parenting, efficient emotion regulation abilities, effective child-rearing practices, etc.) and parental stressors (e.g., poor co-parenting, poor emotion regulation abilities, inconsistent child-rearing practices, etc.)^2^. Hence, when parents’ frail parenting resources chronically fail to compensate for taxing parental stressors, parental burnout may set in by triggering the four core symptoms of this syndrome. First, burnt-out parents experience an intense exhaustion related to their parental role. They feel literally drained out by their role as a parent which they cannot escape from. Second, as a consequence of the first, fatigued parents gradually feel the need to emotionally step back from their children. Third, they feel they are no longer able to thrive in their parenthood and lose all parenting efficacy^3,4^. The fourth and last symptom that characterizes parental burnout is the sharp contrast between the dedicated parent the now burnt-out parent used to be and the minimalist parent he/she has become^5,6^.

For almost a decade now, parental burnout has been of increasing interest to many scholars around the world because both its prevalence and its consequences (for parents and children alike) are alarming. In an international study conducted before the COVID-19 pandemic, Roskam and colleagues^7^ showed that 5 to 9% of Western parents were struggling with parental burnout and the situation has worsened in many countries ever since the pandemic^8^. In terms of the consequences associated with the condition, although both parents and children suffer the deleterious effects, these are expressed at different levels. Hence, while the burnt-out parent tends to be in poor health^9^ and is prone to suicidal thoughts, the child is sometimes exposed to neglectful treatments and/or verbally/physically violent treatments from his/her exhausted parent^3,10^.

Due to the dire consequences associated with parental burnout, much of the research in the field has hastened to understand (better) the antecedents of the syndrome so as to develop both prevention avenues and treatment options for the condition. Initially, research focused on objective life conditions of the parents, that is, sociodemographic predictors (viz. number and age of the children, living surface area, net household income, hours spent with the children, etc.). Unexpectedly, studies accumulated all over the world repeatedly showed that these sociodemographic characteristics only accounted for a small proportion of explained variance in parental burnout^7,11–17^.

The above cited research on parental burnout further investigated other types of antecedents, of which the parent’s emotional intelligence (e.g., the parent’s emotion regulation abilities), parenting factors (e.g., child-rearing practices efficacy) and family functioning factors (e.g., co-parental support). Interestingly, their predictive weight was much higher than that of sociodemographic factors. This raises the question of whether—compared to sociodemographic features—emotional intelligence factors, parenting factors and family functioning factors *objectively* play a greater role in predicting parental burnout than sociodemographic characteristics do, or whether the higher predictive power of emotional intelligence factors, parenting factors and family functioning factors is explained by the fact that they are measured in a way that reflects the subject’s *perception.*

The question of what role perceptions possibly play in parental burnout needs addressing since cognition has been extensively shown to play a crucial role in triggering and/or regulating emotions and in (mental) health^18–21^. What is more, research pertaining to job burnout showed that occupational stress was positively related to job burnout by virtue of cognitive appraisals^22–24^. Additional studies explored the predictive weight of perceived versus objective work overload and it emerged that the former explained significantly more variance in occupational burnout than the latter^25,26^.

As far as parental burnout is concerned, a preliminary study conducted during the COVID-19 global pandemic^27^ showed that cognitive appraisals played both a moderating and a mediating role in parental burnout, thereby suggesting that cognitive appraisals might be operative in parental burnout. Nevertheless, as this study was correlational in design, it precludes any causal interpretation. The present study aims to investigate the causal role of cognitive appraisals in parental burnout through an experimental manipulation of the former. Several studies in the field of emotion and cognitive science more broadly showed that it is possible to manipulate appraisals^28–33^. In the present study, we will use autobiographic memory priming to manipulate parents’ appraisals of three factors known to weigh heavily in predicting parental burnout (i.e., perceived co-parental support, perceived emotional competence abilities while parenting and perceived efficiency of parenting practices. Bivariate correlations between parental burnout and these three factors range from −0.40 to − 0.45^34,35^) and investigate the impact of this between-subject manipulation on pre-post manipulation parental burnout scores. Our hypotheses are as follows:

1. Compared to control subjects, we expect participants assigned to each of the three experimental conditions respectively (i.e., “perceived co-parenting support”, “perceived emotional regulation abilities while parenting” and “perceived efficiency of child-rearing practices”) to display a decrease in parental burnout scores from pre- to post- manipulation measure.
2. We do not necessarily expect any statistically significant difference in parental burnout difference scores between the three experimental conditions (i.e., “perceived co-parenting support”, “perceived emotional regulation abilities while parenting” and “perceived efficiency of child-rearing practices”). Should they emerge, differences between experimental conditions may suggest that some factors are more subject to appraisal manipulation than others, and future studies would then be needed to go deeper into this finding.

## Results

We conducted all statistical analyses using the Statistical Package for the Social Sciences, SPSS version 27^36^.

### Preliminary analyses

Baseline parental burnout scores of the participants across the four conditions were compared using ANOVAs. Analyses confirmed that these scores were statistically equivalent at baseline, *F*(3,309) = 1.18, *p* = 0.32, thereby indicating that the randomization allowed for comparable groups.

Although the assumptions related to the repeated measures ANOVA (independence of the data, normality of the response variable and sphericity) were met, we applied a root square transformation on both baseline and post-experimentation parental burnout variables to increase the normality of their distribution. Since the preregistered main analysis that we performed on both transformed and non-transformed variables yielded similar results, we reported here the results obtained from the original variables in order to facilitate the interpretation of the results.

### Manipulation check analyses

Both the seriousness and the degree of investment put by the participants in the experimentation need underlining. In this respect, we invite the reader to appraise the quality of the data gathered from the experiment in Appendix 1 (made available on OSF) where we give some examples of participants’ answers.

**Figure 1:**
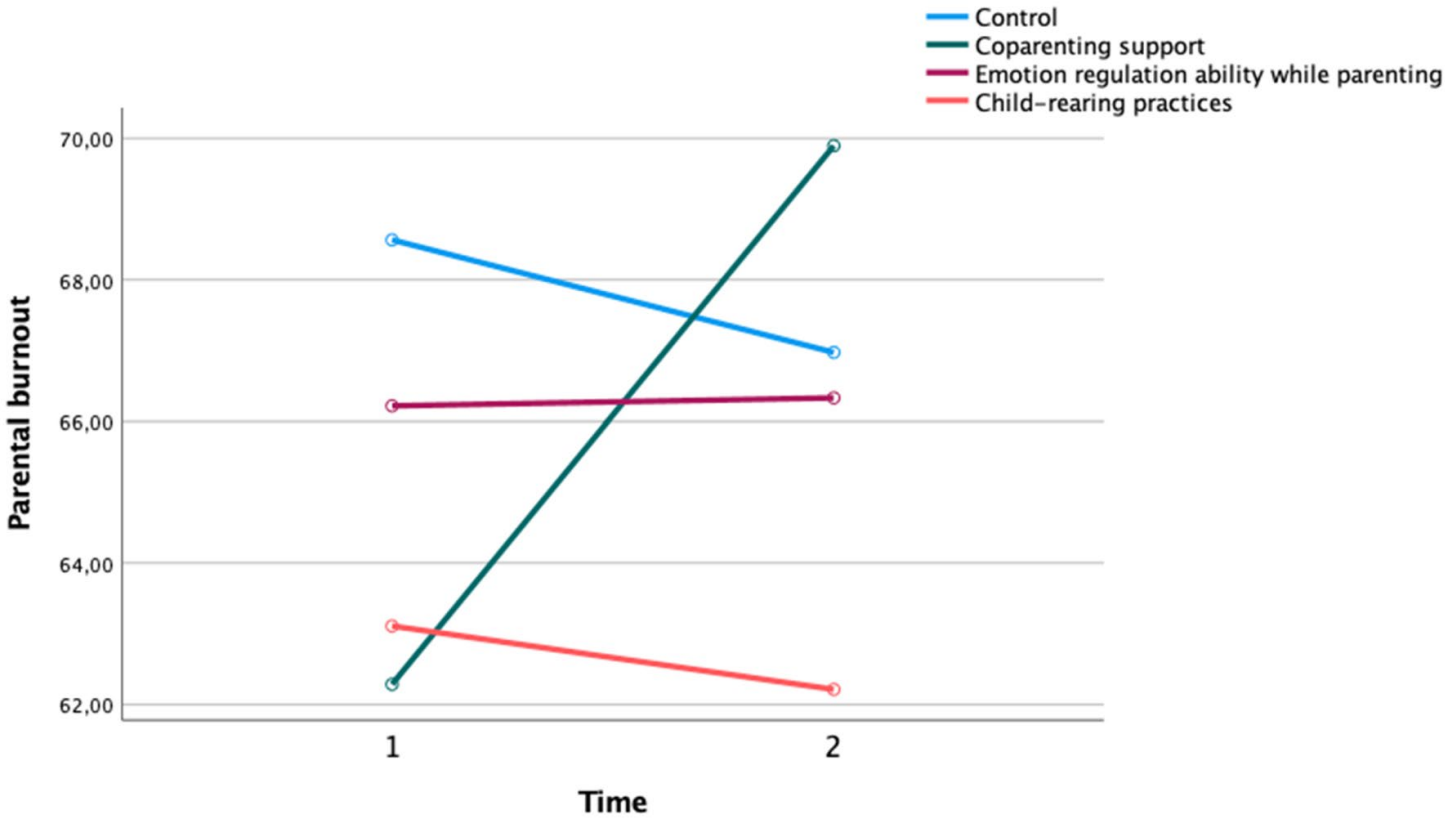
Manipulation check: significant Time × Condition effect for the “co-parenting support” condition. Y axis designates perceived co-parenting support scores, while X axis designates the evolution through the two measurement occasions. “Co-parenting support” condition (*M*_T1_ = 62.29; *SD*_T1_ = 30.05, *M*_T2_ = 69.90; *SD*_T2_ = 26.39), “Emotion regulation abilities while parenting” condition (*M*_T1_ = 66.22; *SD*_T1_ = 27.90, *M*_T2_ = 66.33; *SD*_T2_ = 27.95), “Child-rearing practices efficacy” condition (*M*_T1_ = 63.11; *SD*_T1_ = 33.61, *M*_T2_ = 62.21; *SD*_T2_ = 34.70), “Control” condition (*M*_T1_ = 68.56; *SD*_T1_ = 30.96, *M*_T2_ = 66.97; *SD*_T2_ = 31.14).

Change in the scores of perceived co-parenting support, perceived emotion regulation abilities while parenting and perceived child-rearing practices efficacy between pre- and post- test served as a manipulation check. ANOVAs with repeated measures (pre- and post- manipulation) were run on “perceived co-parenting support”, “perceived emotion regulation abilities while parenting” and on “perceived efficiency of child rearing-practices” conditions separately to verify whether our manipulation of appraisals was effective in each/any of the three experimental conditions. Results showed that only the manipulation of the appraisal of the co-parenting support was effective, with a statistically significant increase of the perceived support received by the co-parent over the two measurement points. Thus, analyses related to the “co-parenting support” condition revealed no effect of Time *F*(1, 280) = 3.23, *p* = 0.07, $\eta p$^2^ = 0.01, but did reveal a significant Time x Condition effect *F*(3, 280) = 9.19, *p* = 0.00, $\eta p$^2^ = 0.09. Figure 1 depicts the above-reported significant interaction effect and presents the means and standard deviations of each condition through the two measurement occasions.

Regarding the “emotion regulation abilities while parenting” condition, we observed a significant effect of Time *F*(1, 302) = 33.20, *p* = 0.00, $\eta p$^2^ = 0.1, but no significant Time x Condition effect *F*(3, 302) = 1.04, *p* = 0.37, $\eta p$^2^ = 0.01. Similarly, in the “child-rearing practices abilities” condition, we obtained a significant effect of Time *F*(1, 306) = 46.80, *p* = 0.00, $\eta p$^2^ = 0.13, but no significant Time x Condition effect *F*(3, 306) = 2.35, *p* = 0.07, $\eta p$^2^ = 0.02.

### Main analysis

**Figure 2:**
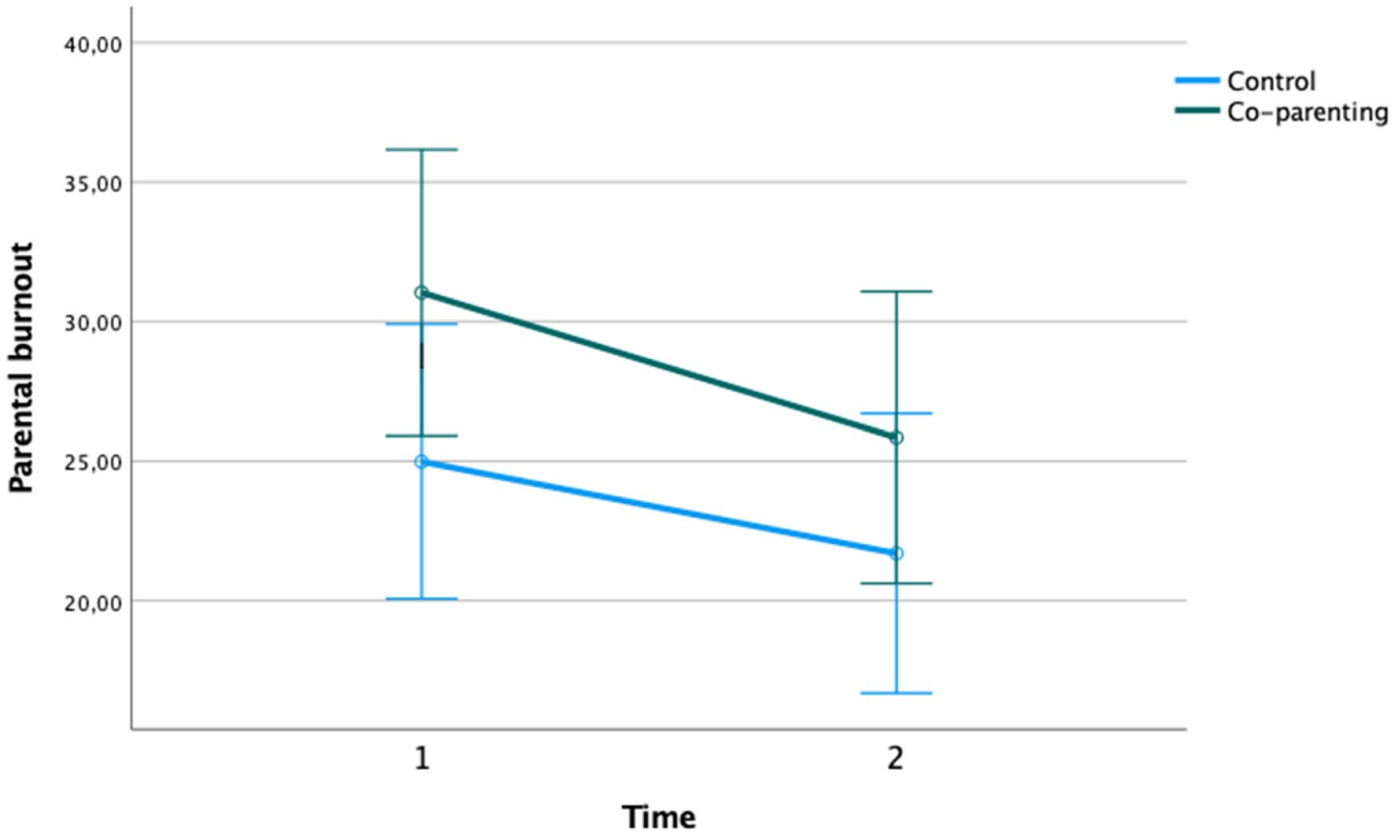
Significant effect of the manipulation of the appraisal of co-parenting support on parental burnout scores pre-post manipulation. Y axis designates parental burnout scores, while X axis designates the evolution across the two measurement points. “Co-parenting support” condition (*M*_T1_ = 31.04; *SD*_T1_ = 24.90, *M*_T2_ = 25.85; *SD*_T2_ = 25.19), Control condition (*M*_T1_ = 24.99; *SD*_T1_ = 20.67, *M*_T2_ = 21.70; *SD*_T2_ = 21.27). Error bars: are ± 2 *SE*.

Insomuch that Manipulation check analyses (presented above) revealed an effect of the “co-parenting support” condition only, we proceeded with the testing of our main hypothesis comparing this experimental condition to the control group (See Preregistration section below). Before conducting the analysis, we compared the baseline parental burnout score of the participants between the two conditions (control versus “co-parenting support condition”) using ANOVAs. Analyses confirmed that the scores were statistically equivalent at baseline, *F*(1,163) = 2.90, *p* = 0.09, $\eta p$^2^ = 0.02. Our hypothesis posited a positive effect of the experimental manipulation, namely, a decrease of parental burnout scores at post-test. Thereupon, to enquire whether, compared to control subjects, participants assigned to the “co-parenting support” condition displayed a decrease in parental burnout scores from pre- to post-manipulation measure, we performed a two-way ANOVA with repeated measures, with Time (parental burnout scores pre–, post– manipulation) as the within-subject factor and Condition (“perceived co-parenting support”, “control”) as the between-subject factor. Analyses showed both a main effect of Time *F*(1, 163) = 119.13, *p* = 0.00, $\eta p$^2^ = 0.42, and an interaction effect of Time × Condition *F*(1, 163) = 5.98, *p* = 0.06, $\eta p$^2^ = 0.04. This significant effect of interaction is illustrated in Fig. 2 whose note presents the means and standard deviations associated to the two conditions across the two measurement times.

## Discussion

The aim of the present study was to investigate the causal role of cognitive appraisals in parental burnout through an experimental manipulation of the former. Dovetailing with recent findings arguing that perceived partner support has a positive impact on parental burnout^37^, our main analysis results suggest that cognitive appraisals have a causative effect on parental burnout since our successfully raising the parents’ perception of their co-parent being supportive resulted in a statistically significant reduction in parental burnout scores compared to the control group. While this important finding opens up promising clinical avenues for partnered parents (which would consist in augmenting the perceived partner parental support to alleviate parental burnout symptoms), it also raises important ethical considerations. Indeed, however appealing this lever for action in reducing parental burnout symptoms may seem *prima facie*, it would be literally unethical to raise a parent’s positive perception of their partner being supportive in the case where the latter is actually (hardly) not. Howbeit, if this result validates our first Hypothesis, it does it partially only since we were unable to derive conclusive results for the “emotion regulation abilities while parenting” condition and the “efficacy of child-rearing practices” condition.

In view of our participants’ dedication to the experimental set-up, the results related to the manipulation check analyses inarguably testify to the complexity of manipulating parents’ cognitive appraisals. As a matter of fact, even though we did not expect any difference in reducing parental burnout scores between the three experimental conditions (i.e., "co-parenting support" condition, "emotion regulation abilities while parenting" condition, and "efficacy of child-rearing practices" condition) (see Hypothesis 2 in Introduction), it was only possible to increase the participants' perception of their co-parent being supportive, which (as expounded above) ultimately resulted in a decrease in parental burnout scores. We surmise that the nature of the manipulation itself pertaining to the “co-parenting support” condition may constitute an explanatory element in successfully raising the perception of the co-parenting support. Indeed, while reporting two positive factual past events of one’s partner offering parental support (insofar as there ever was at least one, of course) is within everyone’s reach, bringing back to mind positive memories of oneself being skillful at managing one’s emotions (“emotion regulation abilities while parenting” condition) or of oneself being able to juggle an effective range of parenting practices (“efficiency of child rearing-practices” condition) may prove more complex since it requires both a certain degree of emotional intelligence and introspective skills^38^.

Furthermore, having participants recall memories which involve the parental dyad might constitute another explanatory element in our manipulating the perceived co-parenting support successfully. In this respect, unlike the two other experimental conditions (i.e., “perceived emotion regulation abilities while parenting” and “perceived efficacy of child rearing practices”) in which we asked the participant to recall a positive past situation involving only themselves and their child, in the successful “co-parenting support” condition, the positive memories brought back to mind necessarily involved a third-party and not any third-party since it was the participant's co-parent. Considering Fredrickson’s research which highlights the critical role co-experienced positive affects play in (amongst others) social bonding and well-being^39,40^, we posit that engaging in a task that consisted in picturing one’s love/life partner being supportive might have triggered strong positive affects which inflated the participant’s perceived support received from their co-parent post-experimentation. As for parents who share fulfilling co-parenthood with a non-loving partner (divorced parents who manage to maintain a good/supportive relationship as part of their co-parenting arrangement, for example), the literature on social support^41^ is further informative, as it highlights the important role played by the parent’s perception of his or her potential to access support from those around them in reducing parental burnout^42^.

Despite randomized group allocation by Qualtrics, parental burnout scores at baseline differed between control and “co-parenting support” conditions. Although ANOVAs (see “Main analysis” section) revealed that this difference was not statistically different, we cannot rule out that this initial difference between the two conditions may have had an impact on the results, since the participants in the “co-parenting support” condition had a greater margin for improvement in reducing their parental burnout scores, as compared to their counterparts in the control group. Nonetheless, we are confident that if this difference impacted the results, it did it very slightly as testifies the small effect size associated with the ANOVAs results ($\eta p$^2^ = 0.02) that showed the equivalence of the scores at baseline.

Another unexpected result showed that the subjects randomly assigned to the control condition in our main analysis also displayed a decrease in their parental burnout score (although not statistically significant) over the two measurement occasions in the absence of any specific manipulation aimed at making this score decrease. As shown by Blanchard et al.^43^, this suggests that parental burnout might fluctuate over time more importantly than we thought. A further attempt at explaining the observed decrease in parental burnout scores through the two measurement occasions in the control group may lie in the optional qualitative comments left by many participants at the end of the study. Many expressed their interest for the experiment and their gratitude for having been enrolled in the study which offered them precious time focusing on their being a parent, away from the daily grind. Hence, the mere fact of “*having me think of myself as a parent*”, to quote one parent’s exact words, likely positively influenced participants’ parental burnout scores post-experimentation, despite the absence of increased perceptions of the co-parenting support received.

Since previously accumulated evidence has shown that parental burnout is associated with more negative and less positive appraisals of one’s parenthood^17,27^, our results (which did not allow us to fully support our main hypothesis of causality) call for very stimulating lines of questioning at the crossroads of clinical and theoretical viewpoints.

From a clinical viewpoint first, we hope that the above shown intricacy in positively altering the nature of parents’ cognitive appraisals will not deter mental health professionals to address the pervasive negative cognitive processes settled in burnt-out parents so as to circumscribe, insofar as possible, the development of a myriad of comorbid mental conditions such as depression and anxiety (to cite but a few), themselves fueled by negative cognitions^44^.

Theoretically, the situation of the distraught father portrayed in Introduction which had led us to hypothesize that cognitive appraisals might be the explanatory factor in the onset and maintenance of parental burnout now raises the question of knowing whether negative appraisals, instead of fueling parental burnout would rather not result from it. To shed light on this newly raised question, empirical research should be conducted to determine whether a decrease in parental burnout scores would be concomitant with more/less positive/negative appraisals of the various dimensions of parenthood under consideration in the present work (viz. perceived co-parenting support, perceived emotion regulation abilities while parenting and perceived efficacy of child rearing practices).

### Limitations and future directions

As we expounded in the Discussion, the tasks related to the “emotion regulation abilities while parenting” condition and the “efficacy of child-rearing practices” condition may be more complex to achieve than the one related to the “co-parenting support” condition since they necessitated skills such as emotional intelligence and introspection. Thus, it might be fruitful to rethink the tasks in such a way that they would be more easily accomplished by all. For example, by remaining more concise and general in the examples that we gave, thus limiting any possibility of disengagement from the task for fear of its complexity^45^.

In addition to a simplification of the tasks, we posit that the manipulation pertaining to the “perceived emotion regulation abilities while parenting” and the “perceived efficacy of child-rearing practices” conditions respectively would gain in effectiveness if they were administered several times (at least two to three times) within a week’s time, thus increasing the chance of success in priming positive autobiographical memories in participants assigned to these two specific conditions.

What is more, and still as regards the experimental conditions in which the manipulation was not effective, while we do not think that the online format of the study did any disservice to the experimentation, it may be further interesting to compare the present study results with those of a study that would be replicated in situ.

Given the equivalence of each experimental group and the control group to each other in terms of socio-demographic variables (see supplementary material), we did not control for socio-demographic variables in our statistical models. This could nevertheless prove interesting for future research, provided the sample size is enlarged to allow satisfactory statistical power.

Last and foremost, although not without flaws, the present study provides an interesting impetus to the young parental burnout area of research as it pioneers experimental methods in this field. Thus far, parental burnout research has undeniably aptly identified many variables relevant to parental burnout but, alas, almost exclusively through correlational studies^35^. Therefore, we hope that our present contribution is just the beginning of a long series of promising experimental work that will reveal causal relationships in parental burnout.

### Conclusive comment

Although manipulating parents' cognitive appraisals proved to be more complex than expected, we managed to augment the partnered parents’ perception of the receipt of parental support from their co-parent. This effective manipulation subsequently accounted for a decrease in parental burnout symptoms, thereby suggesting a causative relation between cognitive appraisals and parental burnout, and more specifically between positive cognitive appraisal of the support received from one’s co-parent to lower one’s parental burnout symptoms. Our research has noteworthy ethical, theoretical and practical implications that will hopefully be echoed in both scholars and clinical practice.

## Method

### Preregistration

We preregistered our objectives, study design, analysis plan and methods on the Open Science Framework (OSF): https://osf.io/bcr6h/?view_only=bab778653564432d982ae0f9e3d46df0. The current article’s supplementary material provides the reader with SPSS (version 27) syntaxes, a comprehensive overview of both examples of genuine answers given by participants (Appendix 1) and the wording of the prompts related to the experimental manipulation (Appendix 2). Note that we slightly updated the original registration (i.e., data collection duration and procedure, reinforcement of the experimental manipulation). A comprehensive list of the changes brought to the original version can be obtained here: https://osf.io/wpd52?view_only=bab778653564432d982ae0f9e3d46df0.

### Participants

A priori power analysis performed on G*Power software (version 3.1.)^46^ estimated an optimal sample size of 280 participants to detect a medium (*d* = 0.50, $\eta p$^2^ = 0.06) effect-size^47,48^. A sample of 313 French-speaking and English-speaking participants composed of 223 mothers (71.2%) and 90 fathers (28.8%) was recruited through social networks, e-mails and word-of-mouth (snowball sample) to serve the purpose of the study conducted on Qualtrics (Provo, UT). Note that due to an insufficient number of participants recruited in Belgium between January 2022 and June 2022, data collection was completed in July 2022 on Prolific (http://www.prolific.co, UK) (*n*_Belgian_sample_ = 163; *n*_Prolific_sample_ = 150). French-speaking participants (snowball sample) did not receive any financial compensation for their participation but were offered a chance to win 200 euros in a lottery. Their English-speaking counterparts were paid according to Prolific's hourly rate the amount of £3.75 for their participation. Our respondents were aged between 22 and 60 years old (*M*_age_ = 41.42; *SD*_age_ = 7.81) and had at least one child between the ages of 3 and 19 still living in the household (M_number_of_children_ = 2.06; SD_number_of_children_ = 0.93; M_age_of_children_ = 10.05; SD_age_of_children_ = 5.38). Amongst them, 76.7% formed a two-parent family, 11.9% were stepparents, 8.9% were single parents and the remaining 2.6% were parents originating from other family configurations. 83.1% reported having a paid work activity. 20.8%, 41.2% and 38% of the sample declared having a net monthly household income comprised below 2500 €, between 2500 and 5000 € or above 5000 € respectively. Table 1 renders a more accurate account of the socio-demographic profile of the participants across the four different conditions after random group allocation by Qualtrics. In an endeavor to keep the effect of selection bias under reasonable control, the study was presented to participants as designed to understand better the factors which lead to fulfillment or exhaustion while parenting. Inclusion criteria included being an adult parent of maximum 60 years old, fluent in French/English and having at least one child aged from 3 to 19 still living in the participant’s household.

**Table 1:** Socio-demographic characteristics of the sample by condition after random group allocation.

Variable	Co-parenting support received * n* = 79		Emotion regulation abilities *n* = 70		Child-rearing practices *n* = 78		Control *n* = 86	
	*M* (*SD*)	%	*M* (*SD*)	%	*M* (*SD*)	%	*M* (*SD*)	%
Sample								
Snowball French-speaking (vs. prolific)		54.43		48.57		48.72		55.81
Women		73.42		71.43		67.95		72.09
Age	41.22 (7.94)		40.34 (7.17)		41.91(7.63)		42.02 (8.35)	
Educational attainment								
High school only		46.84		32.86		43.59		43.02
Bachelor’s degree		27.85		32.86		23.08		25.58
Master’s degree		7.59		8.57		11.54		18.60
Ph.D		17.72		27.71		21.79		12.79
Number of children living in the household	2 (.92)		2.01 (.97)		1.96 (.89)		2.24 (.94)	
Paid professional activity								
Yes (vs. unemployed)		86.08		81.43		82.05		82.56
Net monthly household income								
Less or equal to £ 2500		18.99		17.14		26.92		19.77
Between £ 2500 and £5500		60.76		71.43		60.26		62.79
Above or equal to £5500		20.25		11.43		12.82		17.44
Family structure								
Two-parent family		86.08		74.29		71.79		74.42
Two-parent blended family		3.80		14.29		15.38		13.95
Single-parent family		6.33		10.00		8.97		10.47
Other		3.80		1.43		3.85		1.16

*N* = 313.

### Ethical considerations

Online informed consent was obtained from each participant. The present study was approved by the ethical committee of the UCLouvain in Belgium and was carried out in accordance with the 1964 Helsinki declaration and its later amendments.

### Procedure

**Figure 3:**
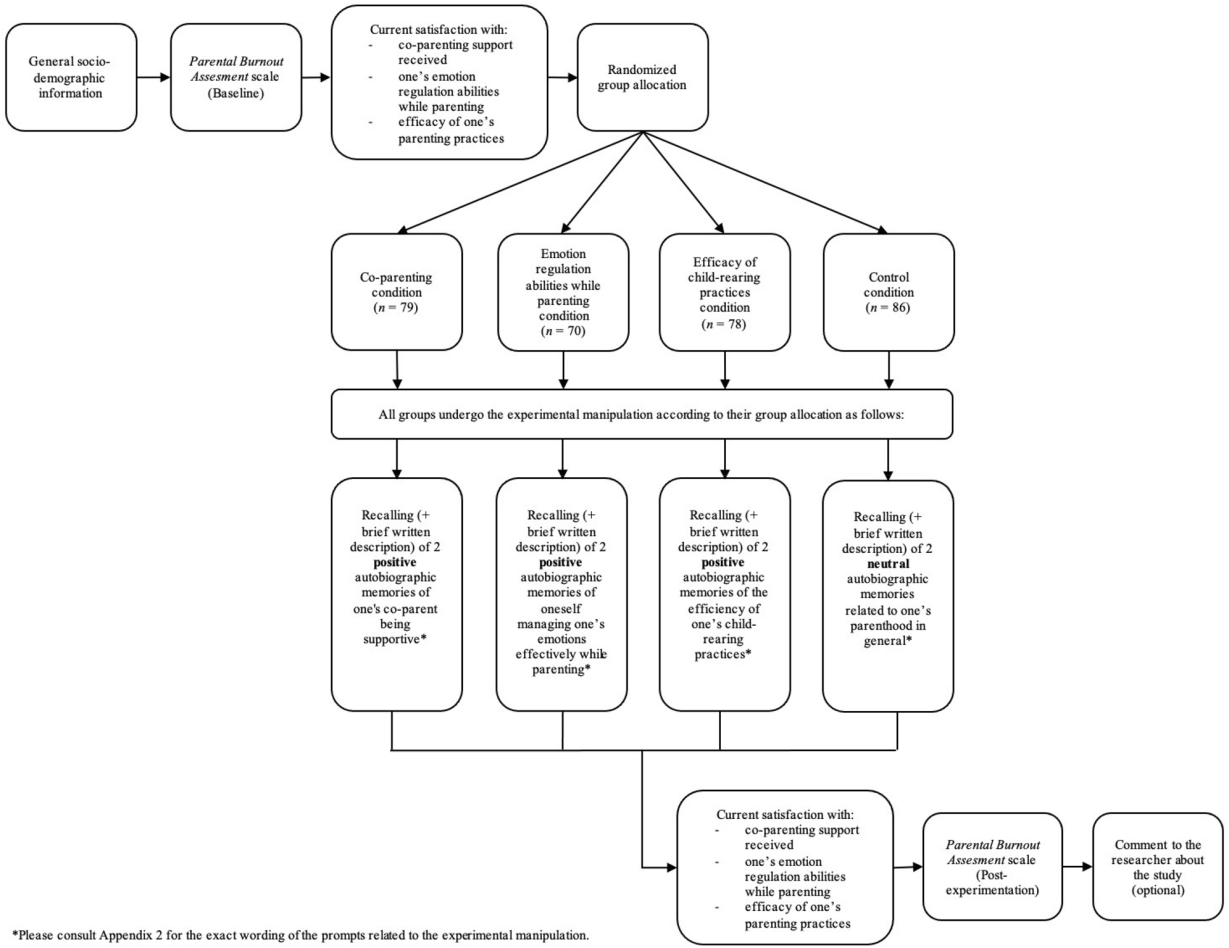
Course of the experiment as experienced by the participants.

Completing the questionnaire took the participant an average of 25 min. In a first instance, we asked participants to communicate general sociodemographic information (see “Measures” section below). Second, we invited parents to complete the *Parental Burnout Assessment* scale^6^ so as to gauge the participant’s level of parental burnout at baseline. Third, we asked a series of questions intended to assess the subject’s personal degree of satisfaction with his/her co-parenting support, his/her emotion regulation abilities while parenting, and the efficacy of his/ her child-rearing practices. In a fourth instance, Qualtrics randomly assigned participants to one of the four conditions, of which three aimed at experimentally manipulating cognitive appraisals by relying on autobiographical positive memories related respectively to either (i) the participant’s perception of the co-parenting support received [condition 1, (*n* = 79)], or to the participant’s perception of his/her emotion regulations abilities while parenting [condition 2, (*n* = 70)], or to the participant’s perception of his/her child-rearing practices efficacy [condition 3, (*n* = 78)]. The last remaining condition consisted in the control group (*n* = 86) in which we asked subjects to remember autobiographical neutral memories about their parenthood. We parameterized Qualtrics so that single parents were randomly assigned to either the control group or to one of the two experimental conditions “emotion regulation abilities while parenting” or “child-rearing practices’ efficacy”, thus strictly avoiding assigning these subjects to the “co-parenting support” condition. In a fifth instance, we asked parents to re-rate their current (at this moment) degree of satisfaction with their perception of the co-parenting support received, their perception of their emotion regulation abilities while parenting and their perception of the efficacy of their child-rearing practices. Finally, in a sixth instance, participants were invited (for a second and last time) to complete the *Parental Burnout Assessment* scale^6^. Note that at the end of the questionnaire, we gave participants the opportunity to leave a comment about the study (optional). Figure 3 visually represents the course of the experiment as experienced by each participant.

In the interests of data quality, we included one attention check item in each measurement occasions of the *Parental Burnout Assessment* (at baseline and post-experimentation). Any participant who failed to meet the 2/2 score for these verification questions was removed from our dataset (46 subjects were removed from the original French-speaking snowball sample only). What is more, a forced option was set on Qualtrics, thereby ensuring a data set deprived of missing data.

### Design

A 4 (Condition: control, perceived co-parenting support, perceived emotion regulation abilities, perceived efficiency of child-rearing practices) × 2 (Time: pre-, post- manipulation) mixed-design was employed, with Condition as the between-subject factor and Time as the within-subject factor.

### Measures

Table 2 presents the bivariate correlations between the measures taken in the scope of the current study.

**Table 2:** Bivariate correlations between the participant’s difference scores of parental burnout, of his/her perception of the co-parenting support received, of his/her emotion regulation abilities while parenting, of his/her child-rearing practices’ efficacy and his/her demographical information.

	Variable	1	2	3	4	5	6	7	8	9	10	11
1	Difference score for parental burnout	–										
2	Difference score for perceived co-parenting support	−0.05	–									
3	Difference score for emotion regulation abilities while parenting	−0.03	0.15**	–								
4	Difference score for child-rearing practices efficacy	−0.20**	0.13*	0.21**	–							
5	Gender	0.04	−0.04	−0.08	−0.03	–						
6	Age	0.03	^−^0.10	0.06	−0.11	−0.14*	–					
7	Educational attainment	0.00	0.04	0.00	−0.05	−0.12*	0.03	–				
8	Family configuration	0.08	0.04	0.08	−0.11*	−0.07	0.18**	0.04	–			
9	Number of children living in the household	−0.01	0.08	−0.00	−0.01	0.24**	0.04	−0.02	−0.08	–		
10	Paid professional activity	0.10	−0.04	0.01	−0.05	0.02	−0.12*	0.15**	0.03	0.02	–	
11	Net monthly household income	−0.14*	−0.03	0.03	0.05	0.14	0.19**	0.04	−0.09	0.12*	−0.15**	–

*p < 0.05.

**p < 0.01.

***p > 0.001.

#### Sociodemographic questions

The survey probed the following socio-demographic features of the participants: gender, age, country of residence, educational attainment, family configuration (two-parent, single parent, other), number of children living in the household, the age and gender of each child living in the household, working status (employed vs. unemployed), net monthly income of the household.

#### Parental burnout

We measured Parental burnout pre- and post-manipulation with the *Parental Burnout Assessment* (*PBA*^6^), a 23-item self-report questionnaire assessing the four core symptoms of parental burnout, namely, Emotional exhaustion (9 items) (e.g., *I’m so tired out by my role as a parent that sleeping doesn’t seem like enough*), Emotional distancing from one’s children (3 items) (e.g., *I do what I’m supposed to do for my child(ren) but nothing more*), Loss of pleasure in one’s parental role (5 items) (e.g., *I don’t enjoy being with my child(ren)*), and Contrast with previous parental self (6 items) (e.g., *I am ashamed of the parent I have become*). The items of the *PBA* use a Likert scale that ranges from 0 to 6 (viz., never, a few times a year, once a month or less, a few times a month, once a week, a few times a week, every day). Summing every item scores *of the PBA* enables to compute the individual’s parental burnout global score, which theoretically ranges from 0 to 138. The higher the score, the higher the level of parental burnout. Internal consistency of the scale in the current sample (*N* = 313) was excellent both at baseline (*α* = 0.96) and post experimentation (*α* = 0.97).

#### Manipulation check: current (i.e., “at this moment”) perceived co-parenting support, perceived emotion regulation abilities while parenting, perceived efficiency of child rearing-practices

We assessed these factors pre- and post-manipulation by means of 6 items accompanied by a Visual Analogue Scale (henceforth VAS) ranging from 0 to 100 (i.e., from not at all to extremely). Among these 6 items, 2 pertained to the participant’s perception of his/her co-parenting support (i.e., [*You feel that you receive sufficient support from your co-parent]* and [*Are you satisfied with the support you receive from your co-parent*?]), 2 pertained to the participants’ perception of his/her emotion regulation abilities (i.e., [*You feel that you manage to regulate your emotions effectively while parenting*] and [*Are you satisfied with the way you manage to regulate your emotions while parenting*?]) and the last 2 items pertained to the respondent’s perception of his/her child-rearing practices’ efficacy (i.e., [*You feel that your child-rearing practices are effective*] and [*Are you satisfied with your child-rearing practices?]).* Summing the two scores obtained on each of the two VAS pertaining to the three categories of items respectively enabled us to compute: a score of satisfaction with the participant’s perception of his/her co-parenting support, a score of satisfaction with the participant’s perception of his/her emotion regulation abilities while parenting and a score of satisfaction with the participant’s perception of the efficacy of his/her child-rearing practices. Note that we opted for the use of a VAS on which the numerical values were deliberately masked to enable us to detect any tiny effect of the manipulation. Internal consistencies in our sample (*N* = 313) were excellent: *α*_Co-parenting_ at baseline = 0.96 and *α*_Co-parenting_ post experimentation = 0.98; *α*_Emotion regulation abilities_ at baseline = 0.92 and *α*_Emotion regulation abilities_ post experimentation = 0.96; *α*_Child-rearing practices_ at baseline = 0.91 and *α*_Child-rearing practices_ post experimentation = 0.95.

#### Experimental manipulation

We prompted participants to recall self-defining memories while parenting. Depending on the condition to which participants were randomly assigned by Qualtrics, they had to recall and briefly describe either: (i) 2 positive autobiographical memories of their co-parent being supportive (condition 1), or (ii) 2 positive autobiographical memories of themselves successfully regulating their emotion while parenting (condition 2), or (iii) 2 positive autobiographical memories of themselves resorting to efficient child-rearing practices (condition 3), or (iv) 2 neutral autobiographical memories related to their parenthood (control condition). Afterwards, we invited participants assigned to the three experimental conditions only (viz. “perceived co-parenting support”, “perceived emotion regulation abilities while parenting” and “perceived child-rearing practices” conditions) to imagine themselves caught in the middle of a very unpleasant conversation during which their interlocutor deliberately started criticizing either the participant’s coparent’s abilities to take care of the children (in “perceived co-parenting support” condition only), or (ii) the participant’s ability to regulate their emotions while parenting (in “perceived emotion regulations while parenting” condition only), or (iii) the participant’s ability to raise their children (in “perceived child-rearing practices” condition only). By way of response to the verbal assault, we asked participants to list a series of illustrative (and assertive) arguments (1 to 10 and at least 2) which proved their interlocutor otherwise. Note that the exact wording of the prompts used for the experimentation is made available in Appendix 2 on OSF.

### Informed consent

Informed consent was obtained from all participants included in the study.

## Supplementary Information

Supplementary Information.

## Data Availability

The analysis plan, methodology and a description of the dataset was preregistered on the Open Science Framework (OSF). De-identified data, Supplementary Material and SPSS 27/Stata 17 syntaxes used for analyses are shared publicly on OSF at https://osf.io/bcr6h/.
