# Supplementary material for: An experimental manipulation of cognitive appraisals in parental burnout
Source: Sci Rep. 2023 Jul 18;13:11585. doi: 10.1038/s41598-023-38587-8 (PMC10353987; doi:10.1038/s41598-023-38587-8)
Supplement: Supplementary file 1 — Supplementary Information. [file 41598_2023_38587_MOESM1_ESM.docx]

## An experimental manipulation of cognitive appraisals in parental burnout

**Supplementary material**

Results related to the analyses of the equivalence of each experimental group and the control group to each other in terms of socio-demographic variables:

The gender and the age of the parents are similar in both groups: *χ^2^* (1) = .04, *p* = .85, *V* = .02 for gender and *t*(163) = .64, *p* = .53, *d* = 8.16 for age. In both the control and the experimental group, the educational attainment of the parent is equivalent *χ^2^* (5) = 5.31, *p* = .38, *V* = .18. The same applies to the family configuration of the parent *χ^2^* (3) = 7.38, *p* = .06, *V* = .21, their professional activity *χ^2^* (1) = .39, *p* = .54, *V* = .05 and their net monthly household income *χ^2^* (5) = 4.20, *p* = .52, *V* = .16. Also in their respective number of children still living in the household are the parents evenly spread across the two groups: *t*(163) = 1.60, *p* = .11, *d* = .93.

**Appendix 1:** Examples of genuine answers given by the respondents.

We are now going to ask you to remember and briefly describe the content of 2 events that triggered **positive** emotions when your **co-parent was supportive in the context of your parenting situation**:
- The first event should be an example of **concrete** support that she/he gave you.
- The second event should be an example of **emotional** support that he/she gave you.
As you do this exercise, we ask you to allow yourself to feel all the positive emotions that these supportive events gave you at that time, as if you were experiencing them again. Allow yourself to take the time necessary to experience the moment and to be fully immersed in the positive emotions associated with it.

Please recall and briefly describe (in the text box below) a **positive memory** of **concrete** support that your partner offered you in order to ease your parenting situation. (For example, your co-parent offered his/her help to take care of some of the routine journeys to or from daycare/school/extra-curricular activities; your co-parent offered his/her help to take care of some of the household chores; your co-parent invested money in material means to improve the comfort of the household; ...) 

Please briefly describe your **positive memory of concrete support** in the text box below. Allow yourself to take the time necessary to experience the moment again and to be fully immersed in the positive emotions associated with it:

**Examples of answers given by three respondents in the “Co-parenting support” condition:**

Participant 1 (case 156): *“Towards the end of last term, I was very stressed out and couldn't face the idea of picking the kids up from their schools. Even though my partner was busy with work herself, she understood my mood and needs and offered to pick them both up at very short notice. This help and kindness made a big difference to my situation at the time and made me feel like I am not alone.”*

Participant 2 (case 159): *“My partner found and agreed to buy a second-hand changing table for downstairs to help me out without needing to go upstairs or crouch/bend over in the lounge to change the baby as I have a bad back that makes the above difficult and painful at times”.*

Participant 3 (case 165): *“My husband often gets up with the children in the morning so I don't have to, as I am often so tired. He will help them get ready for school and leave me in bed. He will keep them really quiet and then get them off to school so quietly I'm not even woken. I am grateful for this support and feel looked after and that my husband understands I don't sleep well and am more tired in the mornings and find them a struggle. It’s so nice to wake up my own time and feel rested and calm and have moments with myself to prepare for my day without feeling like I have to be dragged into the mundane yet chaotic mornings the children tend to create.”*

Now, please recall and briefly describe (in the text box below) a **positive memory** of **emotional** support that your partner offered you in order to ease your parenting situation. (For example, your co-parent complimented you on your parenting skills; you overheard your co-parent speaking highly of your parenting skills to friends or family; your co-parent supported you in the middle of a mild misunderstanding with your child even though he or she did not share your opinion; ...) 

Please briefly describe your **positive memory of emotional support** in the text box below. Allow yourself to take the time necessary to experience the moment again and to be fully immersed in the positive emotions associated with it:

**Examples of answers given by three respondents in the “Co-parenting support” condition:**

**Participant 1:** *“I enrolled our daughters in summer school at our holiday home in Spain. Even though she appeared indifferent to the idea I overheard my partner telling her parents what a great idea it was and that she appreciated my initiative”.*

**Participant 2:** *“My partner complimented me on settling the baby after a tough time getting her to settle after a feed and off to sleep”.*

**Participant 3:** *“Because my husband is not the biological parent of three of my children, when he moved in with us he got a sudden shock about exactly how much hard work it was to deal with three young children and I remember one time in particular when he said he didn't know how I had sloped so well and for so long on my own. I felt pleased that he had noticed how much time and effort I put into my children and how difficult it had been for me. It was a nice feeling, to be noticed and appreciated.”*

Now, imagine yourself having a small talk with someone you do not particularly appreciate but with whom you feel obliged to communicate. At some point, this person starts criticizing your co-parent and says that he/she is not involved enough in raising your children. Whether that person is right or wrong, you feel that he or she is simply not entitled to meddle in your family business. Whether you are likely to respond to this kind of inappropriate comment or not, we ask you to counter-argue his/her point of view here.

Please, list a series of arguments/examples which illustrate that your co-parent is/can sometimes be supportive when it comes to your children:

**Examples of arguments given by three respondents in the “Co-parenting support” condition:**

**Participant 1:**

**Argument 1:** *“She prioritises the kids over work”.*

**Argument 2:** *“She constantly thinks of fun activities we can do as a family”.*

**Argument 3:** *“She buys all the kids’ clothes”.*

**Participant 2:**

**Argument 1:** *“Manages the washing of school clothes”.*

**Argument 2:** *“Sorts out meal plans”.*

**Argument 3:** *“Sorts out the shopping”.*

**Argument 4:** *“Sorts out the playdates / party schedules”.*

**Argument 5:** *“Does the reading and writing homework with child 1”.*

**Argument 6:** *“Compliments me on jobs well done with children”.*

**Argument 7:** *“Asks for help when needed and thanks me for any help given”.*

**Argument 8:** *“Knows they can rely on me when needed but doesn't always ask for help to give me some time to myself”.*

**Argument 9:** *“Does a share of school pickups even though it’s more difficult for them to do it than me”.*

**Argument 10:** *“Supports my discipline approaches in front of the children and only talks to me about it when they are in bed asleep if we need a team review”.*

**Participant 3:**

**Argument 1:** *“My husband is not my children's biological father and yet has supported them financially even when I have not been able to”.*

**Argument 2:** *“My husband gets up nearly every morning with the children, makes their lunches and sees them off to school”.*

**Argument 3:** *“My husband takes the time to read to our youngest every other day and tucks all of the children in at night”*

**Argument 4:** *“My husband cooks for the children every other day and always tries to create nutritious meals for them even when they don't appreciate it”.*

**Argument 5:** *“My husband drives the children to their clubs and picks them up”.*

**Argument 6:** *“My husband is always available to help the children, from fixing their bikes to anything else they ask for help with”.*

**Argument 7:** *“My husband notices things, like my daughters skirt zip being broken or our son needing new shoes”*

**Argument 8:** *“My husband fixes things around the house and makes it nicer for the children to live in”.*

**Argument 9:** *“My husband puts the children first in all aspects of life”.*

**Argument 10:** *“My husband would go without to ensure the children never have to”.*

**Appendix 2 :** Exact wording of the prompts related to the experimental manipulation.

**Experimental “Co-parenting-support” condition**

We are now going to ask you to recall and briefly describe the content of 2 events which triggered **positive emotions** when your co-parent was supportive in the context of your parenting situation. The first event should be an example of **concrete** support that she/he gave you and the second one should be an example of **emotional** support.

As you do this exercise, we ask you to allow yourself to feel all the positive emotions that these supportive events gave you at that time; as if you were experiencing them again. Allow yourself to take the time necessary to experience the moment and to be fully immersed in the positive emotions associated with it.

Please recall and briefly describe (in the text box below) a **positive** memory of **concrete** support that your partner offered you in order to ease your parenting situation.

*(For example, your co-parent offered his/her help to take care of some of the routine journeys to or from daycare/school/extra-curricular activities; your co-parent offered his/her help to take care of some of the household chores; your co-parent invested money in material means to improve the comfort of the household; ...)*

Example of **positive** **concrete** support received from my co-parent: _________________________________________________________

Now, please recall and briefly describe (in the text box below) a **positive** memory of **emotional** support that your partner offered you in order to ease your parenting situation.

*(For example, your co-parent complimented you on your parenting skills; you overheard your co-parent speaking highly of your parenting skills to friends or family; your co-parent supported you in the middle of a mild misunderstanding with your child even though he or she did not share your opinion; ...)*

As you do this exercise, we ask you to allow yourself to feel all the positive emotions that these supportive events gave you at that time; as if you were experiencing them again. Allow yourself to take the time necessary to experience the moment and to be fully immersed in the positive emotions associated with it.

Example of **positive** **emotional** support received from my co-parent: ________________________________________________________

Now, imagine yourself having a small talk with someone you do not particularly appreciate but with whom you feel obliged to communicate.

At some point, this person starts criticizing your co-parent and says that he/she is not involved enough in raising your children. Whether that person is right or wrong, you feel that he or she is simply not entitled to meddle in your family business. Whether you are likely to respond to this kind of inappropriate comment or not, we ask you to counter-argue his/her point of view here.

Please, list a series of arguments/examples which illustrate that your co-parent is/can sometimes be supportive when it comes to your children:

Argument 1 __________________________________________________

Argument 2 __________________________________________________

Argument 3 __________________________________________________

Argument 4 __________________________________________________

Argument 5 __________________________________________________

Argument 6 __________________________________________________

Argument 7 __________________________________________________

Argument 8 __________________________________________________

Argument 9 __________________________________________________

Argument 10 __________________________________________________

**Experimental “Emotion regulation abilities while parenting” condition:**

We are now going to ask you to recall and briefly describe the content of 2 **positive** situations from your parenthood during which you were proud of having **managed your emotions efficiently** while facing mild parenting challenges.

As you do this exercise, we ask you to allow yourself to feel all the positive emotions that this successful emotional management gave you at that time; as if you were experiencing them again. Allow yourself to take the time necessary to experience the moment and to be fully immersed in the positive emotions associated with it.

Please recall and briefly describe (in the text box below) the content of a first **positive** memory of your parenthood during which you were proud of having **efficiently managed your emotions** while facing a mild parenting challenge.

*(For example: your young child was preparing for his or her first school trip abroad and you managed to keep your sadness or anxiety under control so that he or she could not detect your sad/anxious mood; you saw your teenager in trouble and you were able to overcome your feelings of helplessness to establish a fruitful dialogue with him/her; you managed not to let your mood be undermined after having stumbled upon the Facebook profile of that moralizing mother who is continuously posting pictures of her 'perfect' family; …).*

Please briefly describe in the text box below your first **positive** memory of you being **able to manage your emotions effectively** while parenting. Allow yourself to take the time necessary to experience the moment again and to be fully immersed in the positive emotions associated with it.

My first **positive** memory of me efficiently managing my emotions while parenting: ___________________________________________

We would like to remind you of the instruction:

Please recall and briefly describe (in the text box below) the content of a second **positive** memory of your parenthood during which you were proud of having **efficiently managed your emotions** while facing a mild parenting challenge.

*(For example: your young child was preparing for his or her first school trip abroad and you managed to keep your sadness or anxiety under control so that he or she could not detect your sad/anxious mood; you saw your teenager in trouble and you were able to overcome your feelings of helplessness to establish a fruitful dialogue with him/her; you managed not to let your mood be undermined after having stumbled upon the Facebook profile of that moralizing mother who is continuously posting pictures of her 'perfect' family; …).*

*Please briefly describe in the text box below your second* ***positive*** *memory of you being* ***able to manage your emotions* effectively** while parenting. Allow yourself to take the time necessary to experience the moment again and to be fully immersed in the positive emotions associated with it.

My second **positive** memory of me efficiently managing my emotions while parenting: ___________________________________________

Now, imagine yourself having a small talk with someone you do not particularly appreciate but with whom you feel obliged to communicate. At some point, this person starts criticizing your abilities to manage your emotions in your parenthood. Whether that person is right or wrong, you feel that he or she is simply not entitled to meddle in your family business. Whether you are likely to respond to this kind of inappropriate comment or not, we ask you to counter-argue his/her point of view here.

Please, list a series of arguments/examples which illustrate that you can manage your emotions efficiently in your parenthood:

Argument 1 __________________________________________________

Argument 2 __________________________________________________

Argument 3 __________________________________________________

Argument 4 __________________________________________________

Argument 5 __________________________________________________

Argument 6 __________________________________________________

Argument 7 __________________________________________________

Argument 8 __________________________________________________

Argument 9 __________________________________________________

Argument 10 __________________________________________________

**Experimental “Efficiency of child-rearing practices” condition:**

We are now going to ask you to recall and briefly describe the content of 2 situations taken from your parenting experience during which you were proud of having **efficiently handled the situation** while confronted with your child/children being slightly disobedient or difficult. As you do this exercise, we ask you to allow yourself to feel all the positive emotions that this efficient management of the situation gave you at that time; as if you were experiencing it again. Allow yourself to take the time necessary to experience the moment and to be fully immersed in the positive emotions associated with it.

Please recall and briefly describe (in the text box below) a first **positive** memory of a situation taken from your parenthood during which you were proud of having **efficiently handled the situation** while confronted with your child/children being slightly disobedient or difficult.

*(For example, your young child had been begging you ever since you had entered the supermarket to buy him/her the latest gadget displayed at the entrance of the shop and thanks to your parenting expertise, you managed to distract his/her attention so that he/she had lost any interest in the toy before you left the store; the cleaning lady was supposed to arrive any minute but your child had spread his toys all over the floor and would not tidy up but you managed to convince him/her to do so by giving him/her a little playful challenge; Your teenager was thoughtful and sad after a small disagreement with his/her best friend and you managed to make him/her smile again by redirecting his/her attention to another subject; …).*

Please briefly describe in the text box below your first **positive** memory. Allow yourself to take the time necessary to experience the moment again and to be fully immersed in the positive emotions associated with it.

My first **positive** memory of me **efficiently** **handling the situation while parenting**: ______________________________________________________

We would like to remind you of the instruction:

Please recall and briefly describe (in the text box below) a second **positive** memory of a situation taken from your parenthood during which you were proud of having **efficiently handled the situation** while confronted with your child/children being slightly disobedient or difficult.

*(For example, your young child had been begging you ever since you had entered the supermarket to buy him/her the latest gadget displayed at the entrance of the shop and thanks to your parenting expertise, you managed to distract his/her attention so that he/she had lost any interest in the toy before you left the store; the cleaning lady was supposed to arrive any minute but your child had spread his toys all over the floor and would not tidy up but you managed to convince him/her to do so by giving him/her a little playful challenge; Your teenager was thoughtful and sad after a small disagreement with his/her best friend and you managed to make him/her smile again by redirecting his/her attention to another subject; ...).*

Please briefly describe in the text box below your second **positive** memory. Allow yourself to take the time necessary to experience the moment again and to be fully immersed in the positive emotions associated with it.

My second **positive** memory of me **efficiently** **handling the situation while parenting**: ______________________________________________________

Now, imagine yourself having a small talk with someone you do not particularly appreciate but with whom you feel obliged to communicate. At some point, this person starts having doubts about the efficiency of your child-rearing practices. Whether that person is right or wrong, you feel that he or she is simply not entitled to meddle in your family business. Whether you are likely to respond to this kind of inappropriate comment or not, we ask you to counter-argue his/her point of view here.

Please, list a series of arguments/examples which illustrate that your child-rearing practices are efficient:

Argument 1 __________________________________________________

Argument 2 __________________________________________________

Argument 3 __________________________________________________

Argument 4 __________________________________________________

Argument 5 __________________________________________________

Argument 6 __________________________________________________

Argument 7 __________________________________________________

Argument 8 __________________________________________________

Argument 9 __________________________________________________

Argument 10 __________________________________________________

**Control condition :**

We are now going to ask you to recall and briefly describe (in the text box below) the content of 2 events taken from your parenting experience which triggered **neutral** emotions while parenting (i.e., neither negative nor positive emotions – just neutral).

*(For example, a commonplace, routine journey on the way back home with your child(ren); a commonplace family meal during which your children neither particularly behave well nor misbehave; a moment of neutral exchange between you and your partner about the placing of an order related to school material; etc.)*

Please briefly describe your first **neutral** memory in the text box below:

Memory 1: _______________________________________________________________

We would like to remind you of the instruction. Please recall and briefly describe (in the text box below) the content of 2 events taken from your parenting experience which triggered **neutral** emotions while parenting (i.e., neither negative nor positive emotions – just neutral).

*(For example, a commonplace, routine journey on the way back home with your child(ren); a commonplace family meal during which your children neither particularly behave well nor misbehave; a moment of neutral exchange between you and your partner about the placing of an order related to school material; etc.)*

Please briefly describe your second **neutral** memory in the text box below:

Memory 2: _______________________________________________________________
